# U.S. and Canadian pharmacists' attitudes, knowledge, and professional practice behaviors toward dietary supplements: a systematic review

**DOI:** 10.1186/1472-6882-6-31

**Published:** 2006-09-19

**Authors:** Della Kwan, Kristine Hirschkorn, Heather Boon

**Affiliations:** 1 Department of Pharmaceutical Sciences, Leslie Dan Faculty of Pharmacy, University of Toronto, 144 College Street, Toronto, Ontario, M5S 3M2, Canada

## Abstract

**Background:**

Although dietary supplements (DS) are widely sold in pharmacies, the legal, ethical, and practice responsibilities of pharmacists with respect to these products have not been well defined. This systematic review of pharmacists' attitudes, knowledge, and professional practice behaviours toward DS is intended to inform pharmacy regulators' and educators' decision making around this topic.

**Methods:**

Eligible studies were identified through a systematic database search for all available years through to March 2006. Articles were analyzed for this review if they included survey data on U.S. or Canadian pharmacists' attitudes, knowledge, or professional practice behaviors toward DS published in 1990 or later.

**Results:**

Due to the heterogeneity of the data, it was not possible to draw a conclusion with respect to pharmacists' general attitudes toward DS. Approximately equal numbers of pharmacists report positive as well as negative attitudes about the safety and efficacy of DS. There is strong agreement among pharmacists for the need to have additional training on DS, increased regulation of DS, and quality information on DS. In addition, survey data indicate that pharmacists do not perceive their knowledge of DS to be adequate and that pharmacists do not routinely document, monitor, or inquire about patients' use of DS. Despite this, a large proportion of pharmacists reported receiving questions about DS from patients and other health care practitioners.

**Conclusion:**

Further research is needed to explore the factors that influence pharmacists' beliefs and attitudes about DS, to accurately evaluate pharmacists' knowledge of DS, and to uncover the reasons why pharmacists do not routinely document, monitor, or inquire about patients' use of DS.

## Background

Dietary supplements and natural health products are increasingly drawing the attention of regulators, researchers, and health professionals due to high levels of consumption in the U.S. and Canada. This systematic review of pharmacists' attitudes, knowledge, and professional practice behaviors with respect to these products is intended to inform pharmacy regulators' and educators' decision making around this topic.

Complementary and alternative medicine (CAM) is a group of diverse medical and health care systems, practices, and products that are not presently considered to be part of conventional medicine [1]. Dietary supplements are a subset of CAM. In the U.S., the Dietary Supplement Health and Education Act of 1994 (DSHEA) defines a dietary supplement (DS) as "a product (other than tobacco) that is intended to supplement the diet that bears or contains one or more of the following dietary ingredients: a vitamin, a mineral, an herb or other botanical, an amino acid, a dietary substance for use by man to supplement the diet by increasing the total daily intake, or a concentrate, metabolite, constituent, extract, or combinations of these ingredients" [2].

In Canada, these products are classified as natural health products (NHPs) [3]. Under the Natural Health Products Regulations, a NHP is defined as a natural source "substance that is manufactured, sold or represented for use in: (a) the diagnosis, treatment, mitigation or prevention of a disease, disorder or abnormal physical state or its symptoms in humans; (b) restoring or correcting organic functions in humans; or (c) modifying organic functions in humans, such as modifying those functions in a manner that maintains or promotes health" (p. 1573) [3]. Products that fall within this category include herbal remedies, homeopathic medicines, vitamins, minerals, traditional medicines, probiotics, amino acids, and essential fatty acids [3]. Tobacco, marijuana and biologics (e.g., blood-based products, insulin) are excluded [3]. For the purposes of this systematic review, the term dietary supplements (DS) will be used to represent these products.

In recent years, self-medication with DS has become very popular. One U.S. national survey reported a 380% increase in the use of herbal remedies and a 130% increase in high-dose vitamin use between 1990 and 1997 [4]. In Canada, the 2005 Baseline Natural Health Products Survey found that seven in ten Canadians (71%) have used an NHP at least once [5]. Of this group, approximately one-third reported that they do so on a daily basis (38%), followed by "only during certain seasons" (37%), and weekly usage (11%) [5].

The majority of DS sold in the U.S. and Canada are purchased in pharmacies. Approximately half of the adults polled in one U.S. study preferred to purchase alternative health care products from a pharmacy [6]. Similarly, surveys by the Canadian Nonprescription Drug Manufacturers Association (NDMAC) found that 60% of the consumers who buy herbals will purchase them in a drugstore [7].

It has been argued that pharmacists could be instrumental in helping patients make safe and informed choices about DS. Since pharmacists are readily accessible to patients at the point where they are making decisions about purchasing DS, pharmacists are in a good position to provide patients with evidence-based information about DS, especially regarding potential interactions with conventional medications [8]. Pharmacists also have the knowledge and experience to help patients determine when self-medication is appropriate and when the expertise of another healthcare provider is needed [8]. Training in pathophysiology and pharmacology provides pharmacists with the necessary background to interpret and evaluate studies of DS and theoretically places them in an excellent position to determine if a DS is a safe and appropriate option, given any other medication(s) a patient may be taking [8].

Moreover, there is evidence to show that patients view pharmacists as trustworthy and knowledgeable about DS. For example, in one U.S. study, 37% of the respondents agreed that pharmacists' advice is important for alternative therapies and 30% relied on pharmacists for herbal choices [6]. Similarly, the Baseline Natural Health Products Survey found that 43% of Canadians are most likely to say that they completely trust the NHP information provided by their pharmacists [5]. In addition, 18% of Canadians identified pharmacists as primary sources of information on NHPs [5].

Recognizing that pharmacists have an opportunity to develop a unique and credible professional role for themselves as expert advisers on DS, professional associations, such as the American College of Clinical Pharmacy (ACCP), the American Society of Health-System Pharmacists (ASHP), and the Canadian Society of Hospital Pharmacists (CSHP), have recommended that the profession of pharmacy actively embrace DS as part of the pharmacist's scope of practice. The authors of the ACCP's *White Paper on Herbal Products *argue, for example, that ''the basis for pharmacist involvement with herbal products is an extension of their established roles in pharmaceutical care, clinical pharmacy practices, and collaborative health care teams'' (p. 883) [9]. The report also suggests that ''the variability in the degree of scientific evidence on efficacy and safety available to support the use of herbal products makes it even more imperative that pharmacists assume an active role in this area of practice'' (p. 883) [9]. Similarly, the ASHP believes that ''the widespread, indiscriminate use of dietary supplements presents substantial risks to public health and that pharmacists have an opportunity and a professional responsibility to reduce those risks'' (p. 1707) [10]. ASHP further urges pharmacists to ''integrate awareness of dietary supplement use into everyday practice'' (p. 1709) [10]. Moreover, an information paper, *The Role of the Pharmacist with Respect to Complementary/Alternative Medicine*, completed by the Alternative Medicine Task Force of the CSHP suggests: ''Our role as pharmacists is to help educate patients about [NHPs] and to guide them to make informed choices. Our goal should be to ensure that patients who choose to use NHPs do so safely'' (p. 183) [11].

To inform the decision making of pharmacy educators and regulators in U.S. and Canada, with respect to the education and development of professional practice standards for pharmacists in the area of DS, a systematic review of U.S. and Canadian surveys of pharmacists' attitudes, knowledge, and professional practice behaviors toward DS was conducted. It was felt that a review of these three areas would provide a good description of what U.S. and Canadian pharmacists currently think, know, and do with respect to DS that would facilitate the identification of areas in need of further research.

## Methods

Eligible studies were identified by searching the following databases for all available years through to March 2006: Cumulative Index to Nursing and Allied Health Literature (CiNahl), Embase, Healthstar, International Pharmaceutical Abstracts, Medline, and Sociological Abstracts. Search terms included: herb, natural health product, mineral, vitamin, supplement, probiotic, botanic, complementary medicine, alternative medicine, complementary health, alternative health, complementary therapy, or alternative therapy (including truncated versions of these search terms) combined with the following: pharmacy, pharmacist, pharmacies, or pharmaceutical. In cases where these individual search terms matched search headings utilized by the databases, the search headings were used instead (for example, CiNahl uses the terms 'natural product' and 'alternative therapies'; thus these search headings were utilized in lieu of the other search terms identified above). Over 800 articles were identified and subsequently collected through this broadly defined search strategy.

These articles were then hand-searched by two authors (DK and KH) to identify those of relevance to this systematic review. Articles were analyzed for this review if they reported survey data on U.S. or Canadian pharmacists' attitudes, knowledge, or professional practice behaviors toward dietary supplements. Dietary supplements were defined according to the Dietary Supplement Health and Education Act of 1994 (DSHEA) described above [2]. Articles written before 1990 were excluded because the survey data were deemed too old to be useful for policy making. Surveys which only report the products that pharmacists most frequently recommended; qualitative studies; editorials, letters, and comments about pharmacy practice and DS were also excluded. The references listed in the included articles were further hand-searched for other surveys that had not been identified by our database search strategy.

## Results

### Description of included studies

Nineteen studies met the inclusion criteria. One survey which assessed healthcare professionals' (i.e., physicians, nurses, pharmacists, and dietitians) knowledge, attitudes, and practices toward herbs and other dietary supplements was excluded because the results were presented for healthcare professionals in general, and so it was not possible to extract useful information about pharmacists [12]. In addition, hard copies of two surveys could not be obtained [13,14]. Table 1 summarizes the studies included in this systematic review [7,15-29].

**Table 1:** Studies included in this Systematic Review

**Year**	**First Author**	**Design**	**Study Participants and Setting**	**Response Rate and Sample Size**	**Outcomes Measured**	**Topic of Discussion**
1990	Nelson, MV [26]	Cross sectional self completed questionnaire	Random sample of hospital and community pharmacists in U.S. (the Detroit, Michigan metropolitan area) and Britain (nation wide)	U.S. pharmacists 19.7% (n = 197) British pharmacists 63.0% (n = 434)	Knowledge and professional practice behaviors	Alternative health approaches (AHA) which included "acupuncture, auriculotherapy, biofeedback, cellular therapy, chelation therapy, chiropractic, cytotoxic testing, faith healing, food allergy, hair analysis, herbal medicine, holistic medicine, homeopathy, hypnosis, iridology, laetrile therapy, megavitamin therapy, orthomolecular psychiatry, osteopathy, and reflexology"
1998	Brown, CM [16]	Cross sectional self completed questionnaire	Random sample of community pharmacists in the State of Texas, U.S.	36.3% (n = 142)	Professional practice behaviors	Alternative therapies which were defined as "those therapies that are not generally taught in pharmacy and medical schools"
1998	Portyansky, E [27]	Cross sectional self completed questionnaire	Convenience sample of community pharmacists in U.S. (nation wide)	45% (n = 400)	Attitudes, knowledge, and professional practice behaviors	Alternative medicine products which were defined to include "herbal preparations, homeopathic products, and nutraceuticals"
1999	Bouldin, AS [15]	Cross sectional self completed questionnaire	Geographically stratified random sample of community pharmacists in U.S. (nation wide)	26.3% (n = 512)	Attitudes and professional practice behaviors	Herbal medicines which were defined as "commercially-prepared herbal products"
2000	Bokma, A [7]	Cross sectional self completed questionnaire	Convenience sample of community pharmacists in Canada (nation wide)	12.2% (n = 366)	Attitudes, knowledge, and professional practice behaviors	Herbals (not explicitly defined)
2000	Chang, ZG [18]	Cross sectional self completed questionnaire	Convenience sample of pharmacists from multiple practice settings (i.e. community, primary care, academia, hospital, long-term care, and other) in Virginia and North Carolina, U.S.	75.6% (n = 164)	Attitudes and knowledge	Herbal medications which were defined as being "derived from a single plant source"
2000	Little, WR [24]	Not reported	Community pharmacists (sampling not reported) in U.S. (exact location not reported)	n = > 200 (response rate not reported)	Attitudes, knowledge, and professional practice behaviors	Herbals (not explicitly defined)
2000	Montbriand, MJ [25]	Cross sectional face-to-face, computer-assisted interviews	Random sample of pharmacists, physicians, and nurses in active practice in Saskatchewan, Canada	Pharmacists 89% (n = 49) Physicians 58% (n = 52) Nurses 88% (n = 52)	Attitudes and professional practice behaviors	Alternative therapies which were defined as "all health-related products or practices initiated or prescribed by the self, family, friends, or an alternative health-care healer"
2001	Howard, N [23]	Cross sectional self completed questionnaire	Convenience sample of pharmacists from multiple practice settings (i.e. community, hospital, long-term care, drug information, and other) from the 1999 American Society of Health-System Pharmacists (ASHP) Midyear Clinical Meeting in Orlando, Florida	Response rate not reported (n = 70)	Professional practice behaviors	Dietary supplements which were defined as "botanicals (herbs) and nutritional supplements (amino acids, hormones, vitamins, and minerals)"
2002	Hamilton, WR [22]	Cross sectional self completed questionnaire	Random sample of pharmacists (practice setting not specified) and convenience sample of entry-level PharmD students enrolled in an elective CAM class in Nebraska, U.S.	Pharmacists 47% (n = 94) PharmD students 100% (n = 35)	Attitudes	Complementary and alternative therapies (not explicitly defined)
2003	Clauson, KA [19]	Cross sectional self completed questionnaire	Census of pharmacists from multiple practice settings (i.e. community, primary care, academia, hospital, long-term care, and other) in Missouri, U.S.	18.2% (n = 534)	Attitudes, knowledge, and professional practice behaviors	Natural products which were defined to include, but not be limited to, "herbs, mega-dose vitamins, minerals, hormones, and other chemical entities used by patients to maintain or improve their health".
2003	Dolder, C [20]	Cross sectional self completed questionnaire	Random sample of pharmacists from multiple practice settings (i.e. community, hospital, long-term care, and other) in California, U.S.	21.4% (n = 428)	Attitudes, knowledge, and professional practice behaviors	Alternative medications which were defined as "any product, including herbal remedies, vitamins, minerals, and natural products, that may be purchased at a health food store, pharmacy, supermarket, or alternative medicine store/magazine for the purpose of self-treatment"
2003	Welna, EM [28]	Cross sectional self completed questionnaire	Random sample of pharmacists from multiple practice settings (i.e. community, hospital, industry, and other) in Minnesota, U.S.	52.4% (n = 533)	Attitudes, knowledge, and professional practice behaviors	Herbal and other natural products (H/NPs) which were defined as "all products of plant, animal, or mineral origin"
2004	Wood, V [29]	Cross sectional self completed questionnaire	Random sample of community pharmacists in Canada (nation wide)	10% (n = 533)	Professional practice behaviors	Herbal remedy (not explicitly defined)
2005	Brown, CM [17]	Cross sectional self completed questionnaire	Random sample of community pharmacists in the State of Texas, U.S.	27.0% (n = 107)	Professional practice behaviors	Complementary and alternative medicine (CAM) which was defined to include "herbal products, vitamins and minerals, homeopathic products, massage, meditation, and others"
2005	Dunn, JD [21]	Longitudinal self completed questionnaire	Convenience sample of community pharmacists in the State of Utah, U.S.	2003 10% (n = 19) 2004 26% (n = 63)	Attitudes and knowledge	Complementary and alternative medicine (CAM) products which were defined as herbal or natural entities

The majority of the studies (13) were conducted in the U.S. [15-21,23,24,26-28] and three in Canada [7,25,29]. There were four national surveys (two U.S. [15,27] and two Canadian [7,29]). The others surveyed pharmacists in one Canadian province or one or more U.S. states [16-23,25,26,28]. Six were surveys of pharmacists from two or more practice settings which included community, primary care, academia, hospital, and long-term care [18-20,23,26,28]; eight primarily focused on community pharmacists [7,15-17,21,24,27,29]; one compared different health care professionals [25]; and one compared pharmacists with pharmacy students [22]. Twelve surveys had a response rate of less than 60% for pharmacists [7,15-17,19-22,26-29]; and one did not report the response rate [23]. One survey did not report the design, exact study location, sampling procedure, or response rate [24].

It is important to note that the topic of discussion was different for each study (e.g., some studies focused on herbals while others asked about alternative therapies). However, each study incorporated some aspect of DS into the questions asked of pharmacists. For the purposes of this review, all survey findings were assumed to represent pharmacists' attitudes, knowledge, and professional practice behaviors toward DS.

### Pharmacists' attitudes toward DS

Five main themes were identified with respect to pharmacists' attitudes, namely, attitudes toward 'DS in general', 'safety and efficacy of DS', 'education', 'regulation of DS', and 'quality and availability of information on DS'.

#### DS in general

There was little consistency (both within and among surveys) with respect to pharmacists' general attitudes toward DS. In one U.S. national survey, it was found that the majority of respondents believed that many alternative medicine products (including DS) can be beneficial to the health of patients and that alternative medicine has merit [27]. By contrast, in another U.S. national survey, 48% of pharmacists agreed that the majority of their colleagues do not accept herbal medicines (20% disagreed and 32% remained neutral) [15]. Fifty-two per cent of pharmacists in that study also agreed that herbs have a high profit potential (9% disagreed and 39% remained neutral) [15]. Despite this, 57% of pharmacists disagreed that carrying herbal medicines may have a negative influence on a pharmacy's image (19% agreed and 24% remained neutral) [15]. The opposite result was found in a study of pharmacists from Nebraska, U.S.; pharmacists tended to believe those alternative therapies offered by them would decrease the public's respect for the profession [22]. The authors were surprised at this finding but could not offer an explanation [22]. In one additional U.S. study, it was found that pharmacists' attitudes toward CAM did not change significantly between the years of 2003 and 2004. Pharmacists in that study were more likely to report feeling that CAM is a good add-on to prescription medications and to have some interest in CAM [21]. In one Canadian study, 63% of pharmacists said they were in favor of some alternative therapies but not others [25].

#### Safety and efficacy of DS

Pharmacists appear to be fairly evenly split on their attitudes toward the safety and efficacy of DS. Two U.S. studies found that approximately 50% of pharmacists believed that DS are not safe [20,28]. In one national survey, 41% of U.S. pharmacists agreed that most herbal medicines have a high degree of placebo effect (22% disagreed and 37% remained neutral) [15]. This result is supported by a more recent survey of pharmacists from Minnesota, U.S. which found that only 19% of pharmacists believed herbal and other natural products are effective [28]. By contrast, 48% of pharmacists from California, U.S. agreed that alternative medications are effective (24% disagreed and 28% remained neutral) [20]. Of note, one U.S. study found that pharmacist opinions of efficacy may depend on the type of herbal product [24].

#### Education

There is a strong agreement among pharmacists for the need to have additional training on DS. One U.S. study found that the majority of pharmacists agreed that continuing education on herbal medication should be mandatory [18]. In another U.S. study which asked pharmacists about the topic areas that they would like to have continuing education on, pharmacists ranked "interactions" (84.5%), "side effects/adverse events" (80.0%), "patient counseling" (71.2%), "therapeutic uses " (68.2%), and "dosing" (59.2%) as "very important" [19]. Written continuing education was also the most popular method to gain knowledge about natural products (70.2%) and the most preferred method for self-education on natural products (61%) [19]. In a national survey, 92% of Canadian pharmacists said they feel the study of herbals should be mandatory in the pharmacy curriculum [7].

#### Regulation of DS

Survey data indicates that the majority of pharmacists would like increased regulation of DS. In one U.S. study, four out of five respondents said they would feel more comfortable making recommendations to patients if the products were regulated by an independent body [27]. The FDA was picked by 80% of the respondents as the No. 1 choice to be the regulatory group [27]. Similarly, in two other U.S. studies, it was found that 75% of pharmacists believed that herbal products should undergo increased regulation [24] and 96% of pharmacists considered the amount of government oversight/regulation for herbal and other natural products to be less than fully adequate, respectively [28]. In one Canadian study, 82% of pharmacists agreed that alternative medicines should be regulated [25].

#### Quality and availability of information on DS

Based on survey findings, it appears that there is a perceived lack of quality information on DS. One U.S. study reported that 95% of pharmacists felt available information on herbal and other natural products was not adequate or only somewhat adequate [28]. Similarly, in a national survey, only 12% of Canadian pharmacists said they are very satisfied with the quality of information available on herbal products [7]. Of note, 96% of U.S. pharmacists in a national survey indicated that they did not feel they had enough information regarding potential interactions involving herbal products [15].

While there is thus general agreement about the need for further education about these products as well as the lack of quality information and appropriate regulation, attitudes toward DS in general and toward safety or efficacy specifically are mixed.

### Pharmacists' knowledge of DS

Surveys of pharmacists' knowledge primarily included measures for 'perceived', as opposed to 'actual' knowledge.

#### Perceived knowledge

Survey data indicates that pharmacists do not feel their knowledge of DS is adequate. In one U.S. study conducted in 1990, just over 50% of the pharmacists said they 'know something about' or 'know a lot about' herbal remedies [26]. Five more recent U.S. studies found similar results; approximately half of the pharmacists considered their knowledge of DS to be somewhat adequate or average, but only a small proportion were very satisfied with their level of knowledge [19-21,27,28]. Of note, in one U.S. study, it was found that pharmacists' knowledge of herbal products varied widely from herbal to herbal. St. John's Wort ranked highest among the herbals included in the survey in terms of familiarity. 80% of pharmacists stated it is potentially dangerous to a person's health when used in combination with some Rx and OTC products. Ma huang (61%) and yohimbe (50%) were noted as second and third [24]. Similarly, in Canada, 45% of pharmacists in one national survey said they do not have adequate knowledge about herbal remedies to make recommendations regarding their use [7].

#### Actual knowledge

Only one U.S. study evaluated pharmacists' actual knowledge of herbal medications. Overall, pharmacists' herbal medication knowledge test scores were low, with an average of <50%. Pharmacists, in general, were more likely to answer correctly about the uses of herbal medications than about adverse effects and cautions. Pharmacists with prior continuing education on herbal medications, or with access to herbal information at their practice site, had a significantly higher mean test score. However, a non-validated instrument was used in this study. Moreover, for the purpose of statistical analysis, the ''I don't know'' responses were incorporated into the number of incorrect answers on the herbal knowledge test. Respondents were instructed to select ''I don't know'' instead of inappropriately guessing an answer. This may have decreased the overall scores in measuring pharmacists' knowledge of herbal medications [18].

Regardless of whether measures for 'perceived' or 'actual' knowledge were used, survey data clearly indicates that there is a lack of knowledge on DS among pharmacists.

### Pharmacists' professional practice behaviors with respect to DS

The four main types of practice behaviors that were examined by the surveys include: pharmacists' propensity to 'document and monitor patients' use of DS', to 'inquire about patients' use of DS', to 'recommend DS to patients', and 'to seek information on DS'. Surveys also asked pharmacists to report how often they perceive themselves to be used by patients and other healthcare providers as an 'information source on DS'.

#### Documenting and monitoring patients' use of DS

Overall, the rate of documenting and monitoring of patients' use of DS by U.S. pharmacists was low. In a 1998 study from Texas, U.S., pharmacists had documented the use of alternative therapies in the patients' pharmacy records in only 11.1% of the cases [16]. Moreover, 45.3% of pharmacists did not monitor patients' use of alternative therapies [16]. A 2005 study of the same population found a similar result; pharmacists documented CAM use by patients in 11.0% of cases and reported monitoring for drug-related problems in 38.4% of users [17]. Similarly, only 24.1% of pharmacists from California, U.S. stated that they routinely maintained a record of alternative medications taken by their patients [20]. Pharmacists who reported receiving any training in alternative medications or worked in inpatient settings were significantly more likely to record alternative medication use [20]. These results are supported by a U.S. national survey which found that only 7% of pharmacists agreed that they add herbs to their patients' profiles [15].

#### Inquiring about patients' use of DS

Based on survey findings, it appears that not all pharmacists routinely inquire about patients' use of DS. In a 1998 study of pharmacists from Texas, U.S. 35.9% said they never asked any of their patients if they were using alternative therapies [16]. A more recent 2005 study of the same population found a similar result; pharmacists reported that they ''rarely to sometimes'' asked their patients about their use of CAM [17]. By contrast, 51.8% of pharmacists from California, U.S. said that they regularly asked patients about alternative medication use [20]. In two U.S. studies, pharmacists who reported receiving any additional training in alternative medications or CAM were more likely to ask patients about use of these products [17,20]. In one Canadian study, 71% of hospital pharmacists said they asked their patients about alternatives versus 29% of community pharmacists [25].

#### Recommending DS to patients

Survey data indicate that Canadian pharmacists are more likely to recommend DS to patients. In one U.S. study that was conducted in 1999, pharmacist recommendations of herbal products were found to occur on average 4.9 times per month; 91% of respondents made recommendations 10 times or fewer, with 164 respondents making no recommendations at all [15]. Two more recent U.S. studies found that 40 and 56% of pharmacists reported making recommendations to patients on dietary supplements or herbal and other natural products, respectively [23,28]. In addition, 86% of U.S. pharmacists from a 2003 Californian study responded that they did not recommend alternative medications [20]. Similarly, another U.S. study found that almost 70% of the pharmacists sometimes recommended the customer use an OTC product or speak to their physician about a prescription drug instead of using an herbal remedy [24]. By contrast, in one Canadian study, 43% of hospital pharmacists suggested use of alternatives versus 76% of community pharmacists [25]. In comparison to other provinces in Canada, pharmacists from Quebec were found to be most likely to offer patients a choice between a conventional OTC product and an herbal remedy in another study [29].

#### Seeking information on DS

A majority of U.S. and Canadian pharmacists said that they have actively sought information about DS [15,19,25]. For example, in one U.S. study, 79.8% of respondents said they made efforts to learn about natural products one to six times per year [19]. Similarly, in one Canadian study, 75% of pharmacists said they have looked for information about alternative medicines [25].

#### Pharmacists as information sources

A large proportion of U.S. and Canadian pharmacists reported receiving questions about DS from patients and other health care practitioners [7,19,20,24,27,28]. Of note in one U.S. study, 98% of community pharmacists and 58% of hospital pharmacists reported fielding questions from patients about herbal and other natural products [28]. In one Canadian study, 57% of community pharmacists reported being asked about herbal products several times a day and a further 15% are asked at least once a day [7].

Across surveys, it appears that pharmacists do not routinely document, monitor, or inquire about patients' use of DS. U.S. pharmacists are also less likely than Canadian pharmacists to recommend DS to patients. In addition, a large proportion of pharmacists reported actively seeking information on DS and receiving questions about DS from patients and other healthcare providers.

## Discussion

### Pharmacists' attitudes toward DS

Due to the heterogeneity of the data, it was not possible to draw a conclusion about pharmacists' attitudes toward DS in general. However, it appears that U.S. and Canadian pharmacists were fairly evenly split (half positive and half negative) on their attitudes toward the safety and efficacy of DS. These mixed findings may be the result of pharmacists not possessing, or perceiving the availability of, sufficient or consistent information on DS. Without information, understanding of these products is limited, as is confidence about their safety and efficacy. These findings could also be a product of the inconsistent definitions and categorizations of DS utilized across surveys. Of note, the results from one U.S. study suggest that pharmacist opinions of efficacy may depend on the type of herbal product [24].

In contrast, across surveys it is evident that there was a perceived lack of government oversight/regulation for DS. This may be related to the perceived shortcomings of the current U.S. federal regulations on dietary supplements. DSHEA does not require FDA to review evidence of the efficacy or safety of dietary supplements, so manufacturers have no burden to prove that their products are effective or safe [10]. Moreover, DSHEA does not require prospective testing to ensure safety [10]. To remove a product from the market, FDA must prove that the product is unsafe [10]. Under DSHEA, some dietary supplements that were banned from the U.S. market because of concern about their safety have been allowed to return (e.g., sassafras tea, dehydroepiandrosterone) [10]. The Canadian surveys were conducted before the introduction of the Natural Health Products Regulations which likely explains why pharmacists felt there was a need for regulation. Under the new regulations, all NHPs will require a product license before they can be sold in Canada [3]. Approval for sale will be granted by Health Canada based on several tiers of evidence of safety and efficacy [3]. Whether these regulations will positively affect pharmacists' attitudes toward these products remains to be seen.

In addition, there appears to be a perceived lack of quality information on DS. This may reflect the general lack of scientific information available on these products and/or pharmacists' lack of knowledge or access to that which does exist. Further research is needed to reveal the factors that influence pharmacists' beliefs and attitudes about DS. It is also likely that their beliefs and attitudes will differ depending on the type of DS.

### Pharmacists' knowledge of DS

Survey data indicate that pharmacists do not perceive their knowledge of DS to be adequate and that a majority of pharmacists would like to receive additional training on DS, especially in the areas of interactions, side effects/adverse events, patient counseling, therapeutic uses, and dosing [19]. Of note, one U.S. study found that pharmacists' knowledge of herbal products varied widely from herbal to herbal [24]. These findings may be related to the substantial diversity in the extent of DS education at schools of pharmacy. In a survey to describe U.S. pharmacy school curriculum offerings in the areas of natural products and complementary/alternative medicine, it was found that most schools were not providing natural product content in a formalized manner [30]. In most cases, natural products were taught in an elective course [30]. Among the included studies, only one U.S. study evaluated pharmacists' actual knowledge of herbal medications [18]. However, that study had several methodological limitations including the use of a nonvalidated instrument [18].

It is clear that further research is needed to accurately evaluate pharmacists' knowledge of DS in order to determine whether future continuing education programs and courses should be targeted at the introductory or intermediate level. Moreover, such research would enable the identification of topic areas that pharmacists require the most training in.

### Pharmacists' professional practice behaviors with respect to DS

Based on survey findings, it appears that U.S. and Canadian pharmacists do not routinely document, monitor, or inquire about patients' use of DS. The pharmacists who were more likely to ask patients about or record alternative medication use were the ones who worked in inpatient settings or reported receiving additional training in alternative medications or CAM [17,20,25]. None of the surveys asked pharmacists to provide explanations for their behavior. One reason might be the lack of professional practice standards which require pharmacists to routinely document, monitor, or inquire about patients' use of DS. Another reason could be related to the lack of time during consultations to document. In any case, further research is needed to uncover the reasons behind the reported behaviors in these survey findings. This information will be crucial to pharmacy regulators in U.S. and Canada who are interested in developing professional practice standards for pharmacists in the area of DS.

In addition, many pharmacists reported recommending DS to patients [23,25,28] and receiving questions about DS from patients and other healthcare practitioners [7,19,20,28]. Pharmacists appear to be becoming an increasingly important source of information on such products, which appears to be at odds with their assessment of their knowledge of these products.

### Limitations of the review

The main limitation of this review is that all survey findings were assumed to represent pharmacists' attitudes, knowledge, and professional practice behaviors toward DS even though the topic of discussion was different for each study. This was deemed appropriate since each study incorporated some aspect of DS into its topic of discussion. Nevertheless, the generalizations made may not be an accurate representation of what U.S. and Canadian pharmacists think, know, and do with respect to DS as a distinctive category.

The heterogeneity of the included studies also reduced the ability of the review to summarize key trends. Studies were conducted in different parts of U.S. and Canada and at different points in time. Furthermore, studies differed in the design of the response scales, including the wording of the items, and combined results for pharmacists from a variety of practice settings (i.e., community, primary care, academia, hospital, and long-term care). In addition, twelve surveys had a response rate of less than 60% for pharmacists [7,15-17,19-22,26-29] which limits the generalizability of the results. The heterogeneity of the included studies may also explain why there were inconsistencies in the survey findings.

## Conclusion

It is evident from this systematic review that there is a need for pharmacists to receive additional training in the area of DS. Emphasis should be placed on teaching pharmacists how to critically evaluate the use, efficacy, and safety of common DS (including how to identify potential adverse effects and drug interactions) as well as how to interact with patients to address these and other issues associated with common DS. In addition, future surveys should focus on assessing the opinions and behaviors of pharmacists with respect to specific types of DS in order to better facilitate comparisons and generalizations. This information will be crucial to pharmacy educators and regulators in U.S. and Canada who are interested in implementing changes in education and developing professional practice standards for pharmacists in the area of DS.

## Competing interests

The author(s) declare that they have no competing interests.

## Authors' contributions

DK drafted the manuscript. KH and HB edited the manuscript. All authors read and approved the final manuscript.

## Pre-publication history

The pre-publication history for this paper can be accessed here:


